# Supplementary material for: Four New Benzoylamide Derivatives Isolated from the Seeds of Lepidium apetalum Willd. and Ameliorated LPS-Induced NRK52e Cells via Nrf2/Keap1 Pathway
Source: Molecules. 2022 Jan 22;27(3):722. doi: 10.3390/molecules27030722 (PMC8840667; doi:10.3390/molecules27030722)
Supplement: Supplementary file 1 [file molecules-27-00722-s001.zip › molecules-1570830-supplementary.pdf]

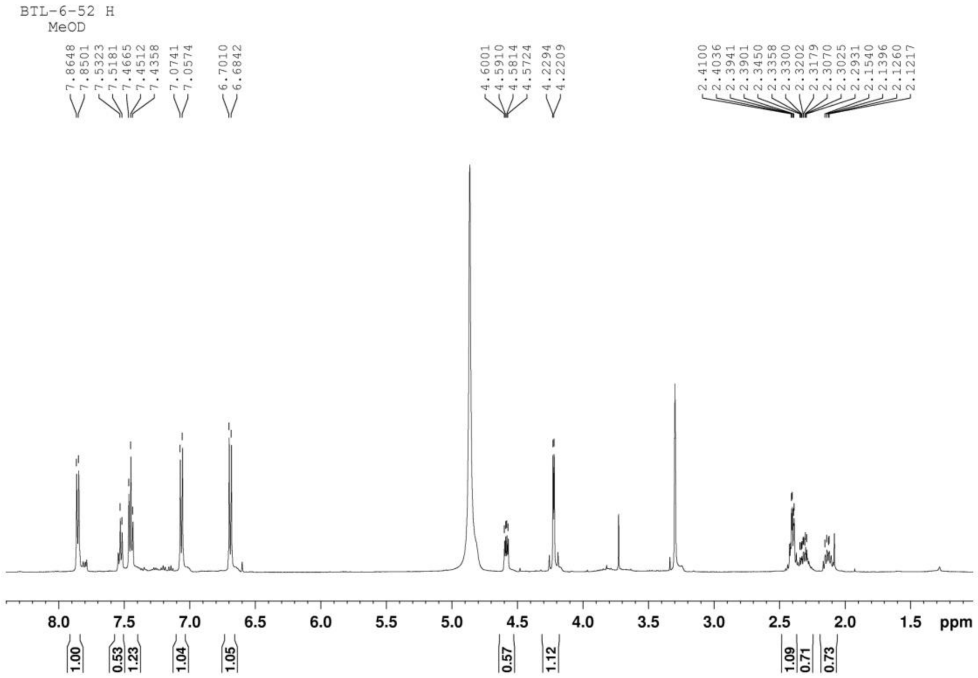

Figure S1 The ^{1}H-NMR spectrum of compound **1**（in CD_3OD）

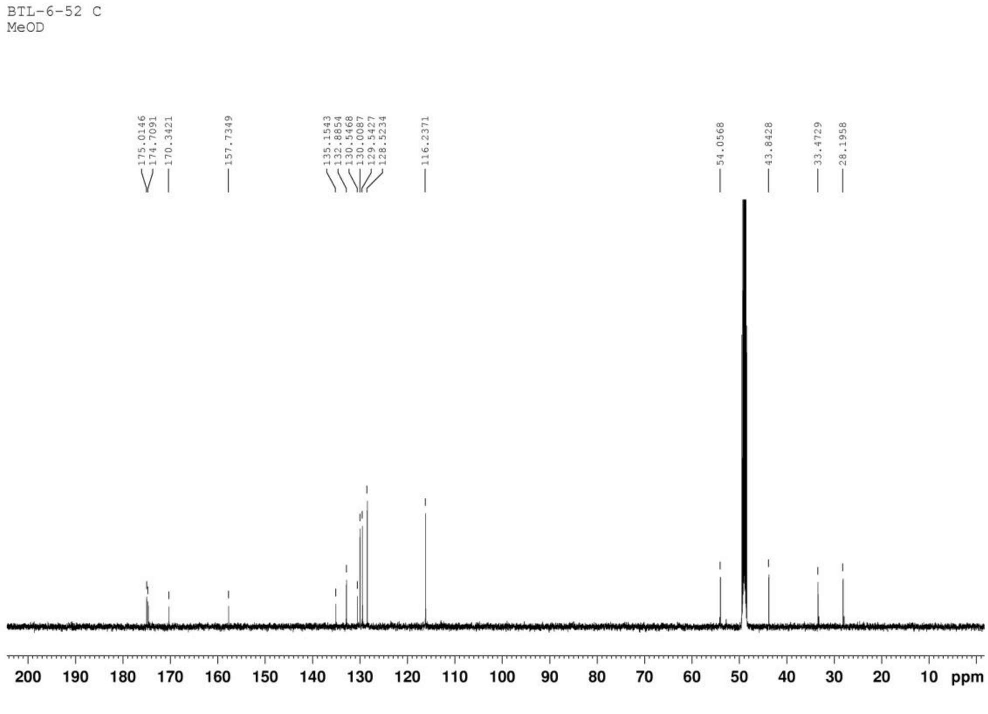

Figure S2 The ^{13}C-NMR spectrum of compound **1**（in CD_3OD）

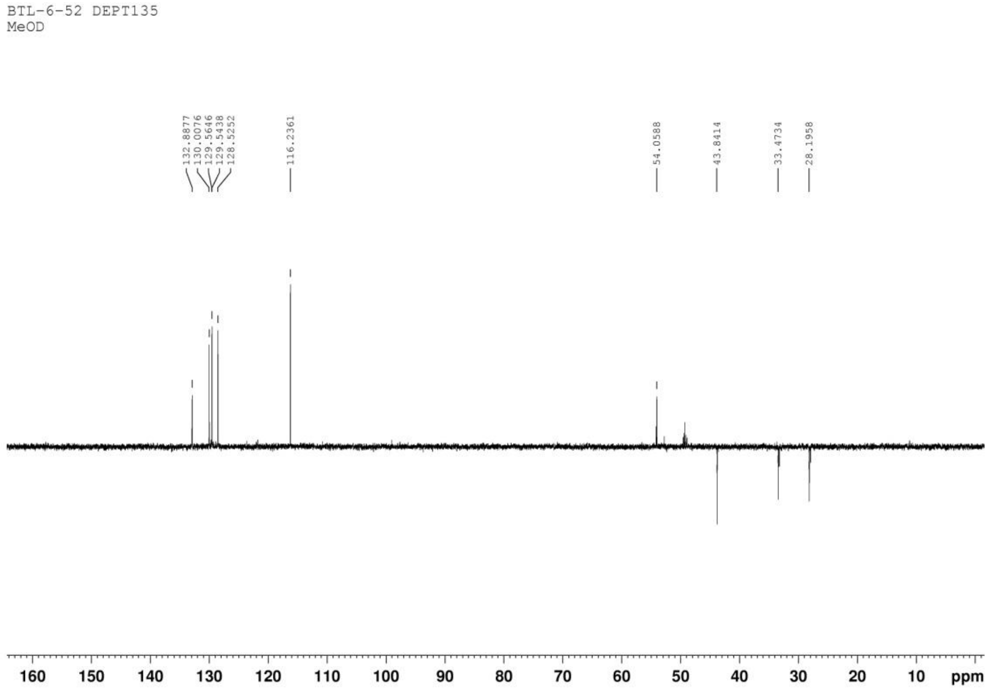

Figure S3 The DEPT 135 spectrum of compound **1**（in CD_3OD）

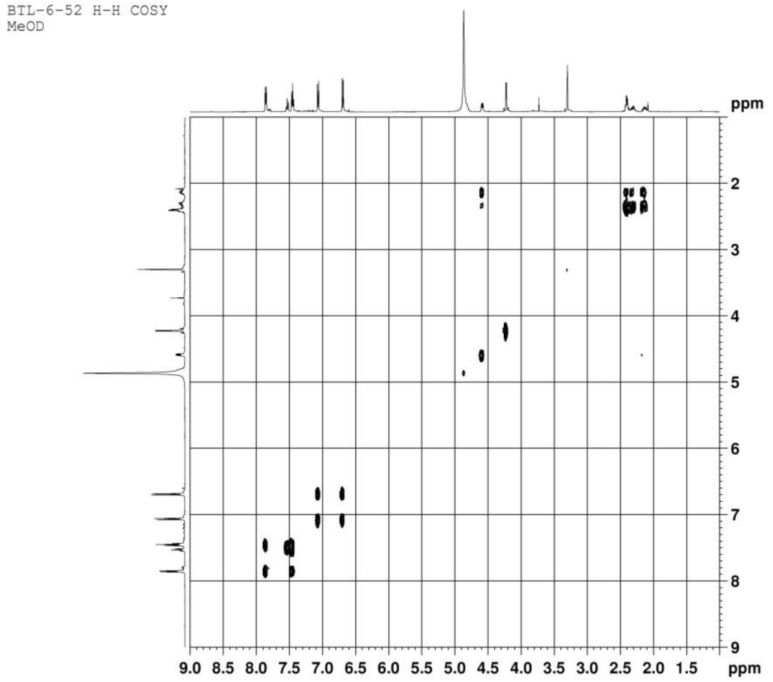

Figure S4 The ^{1}H-^{1}H COSY spectrum of compound **1**

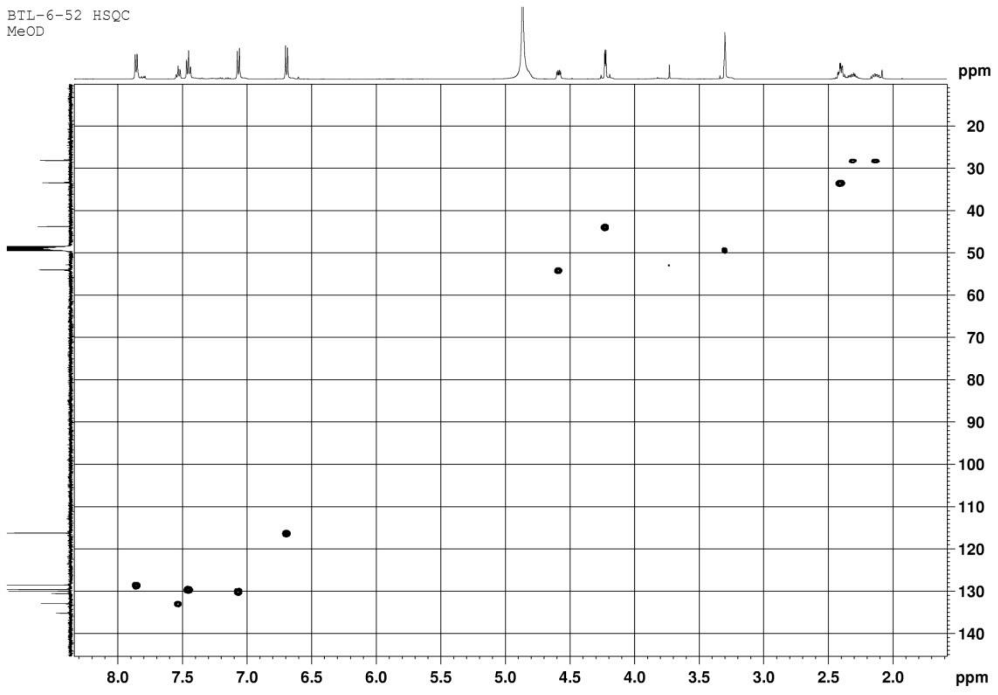

Figure S5 The HSQC spectrum of compound **1**

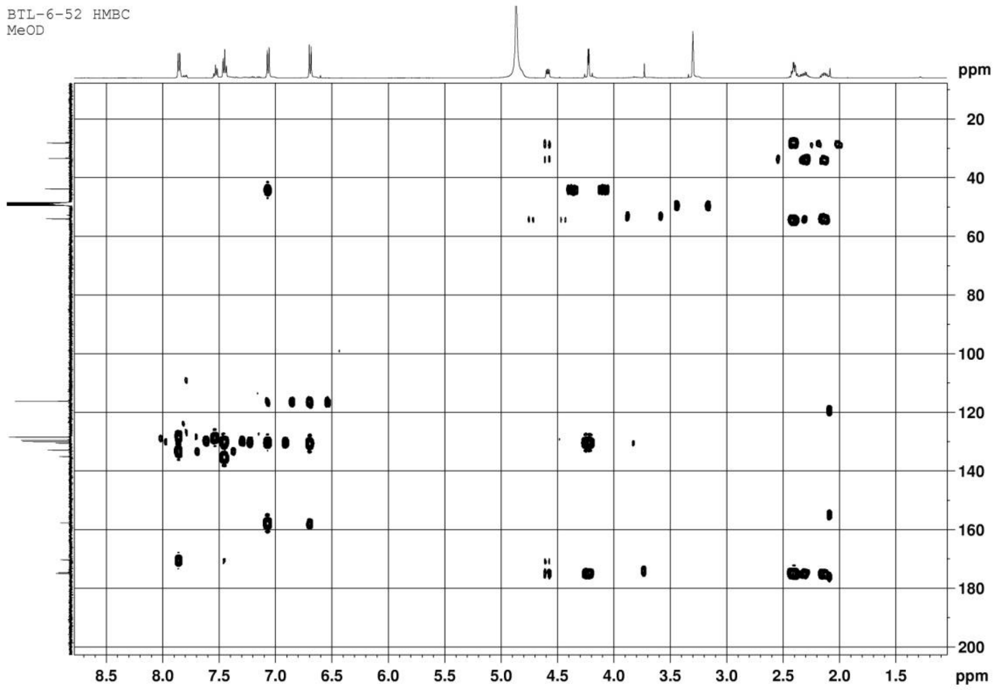

Figure S6 The HMBC spectrum of compound **1**

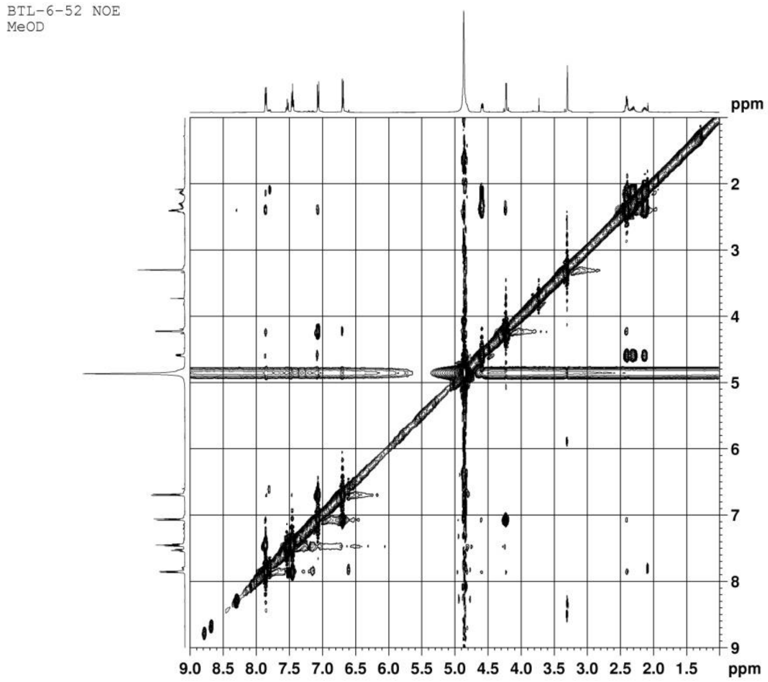

Figure S7 The NOESY spectrum of compound **1**

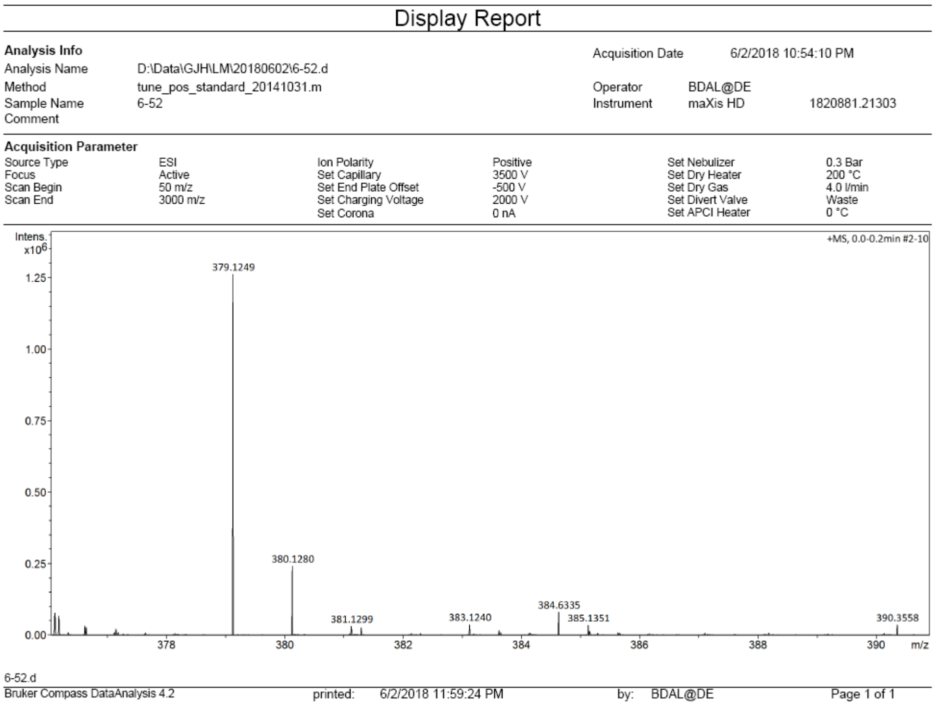

Figure S8 The HRESIMS spectrum of compound **1**

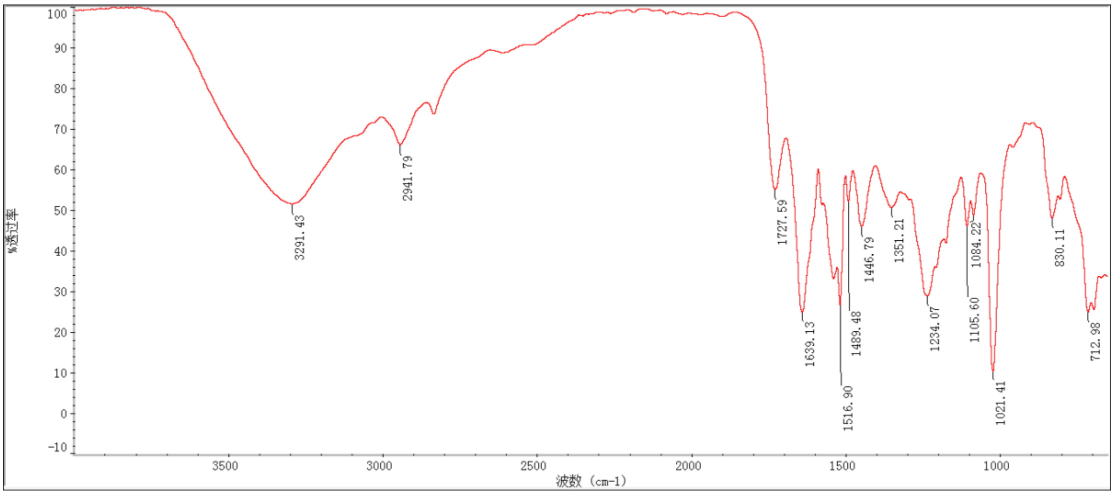

Figure S9 The IR spectrum of compound **1**

Scan Graph

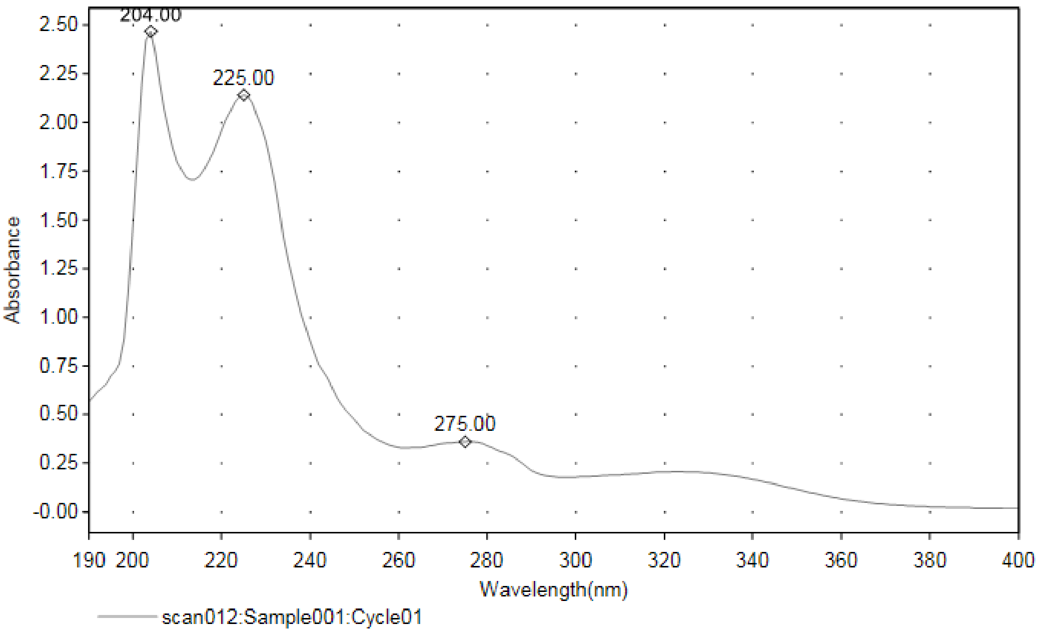

Results Table - scan012,Sample001,Cycle01

nm	A	**Manual Method**
204.00	2.468	Report Values at 3 Wavelengths
225.00	2.142	204.00 nm 225.00 nm 275.00 nm
275.00	.361	Sort By Wavelength

Figure S10 The UV spectrum of compound **1**

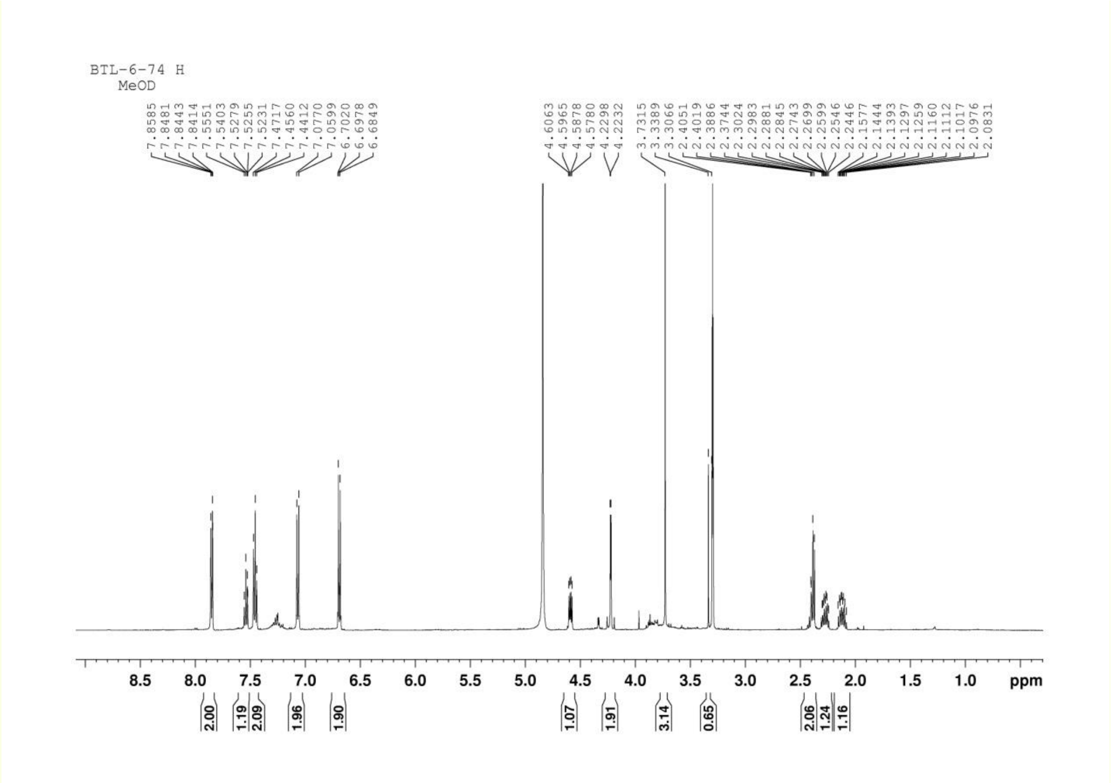

Figure S11 The ^{1}H-NMR spectrum of compound **2**（in CD_3OD）

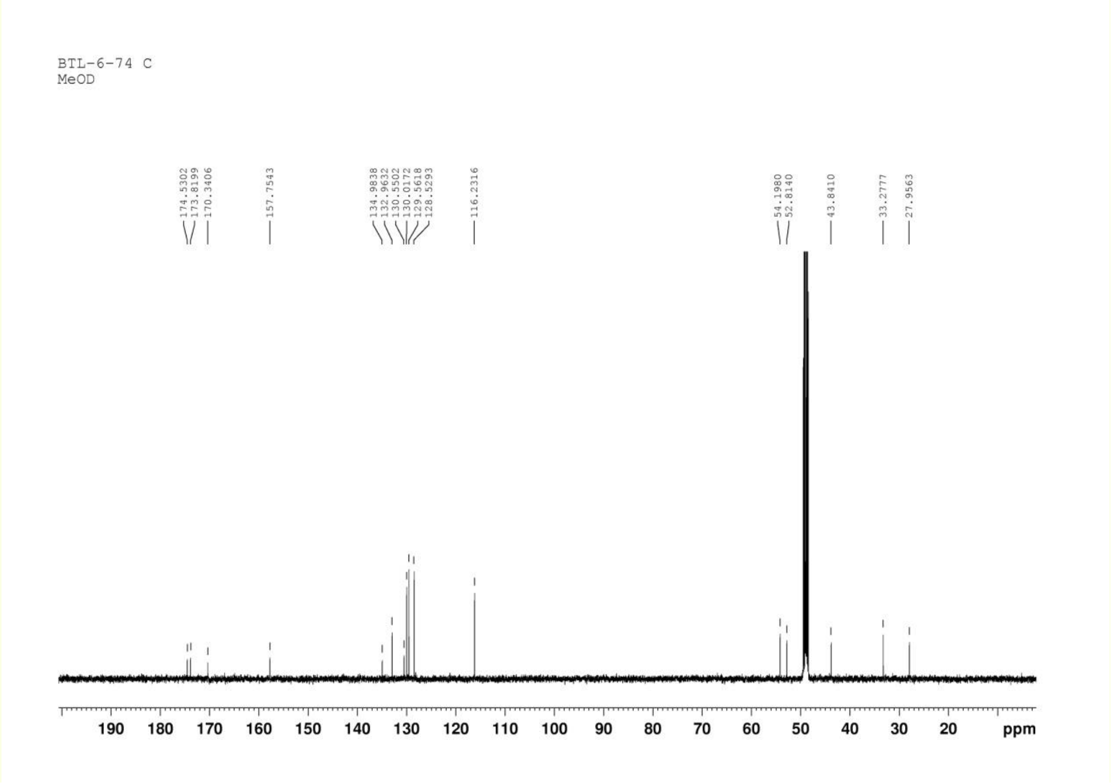

Figure S12 The ^{13}C-NMR spectrum of compound **2**（in CD_3OD）

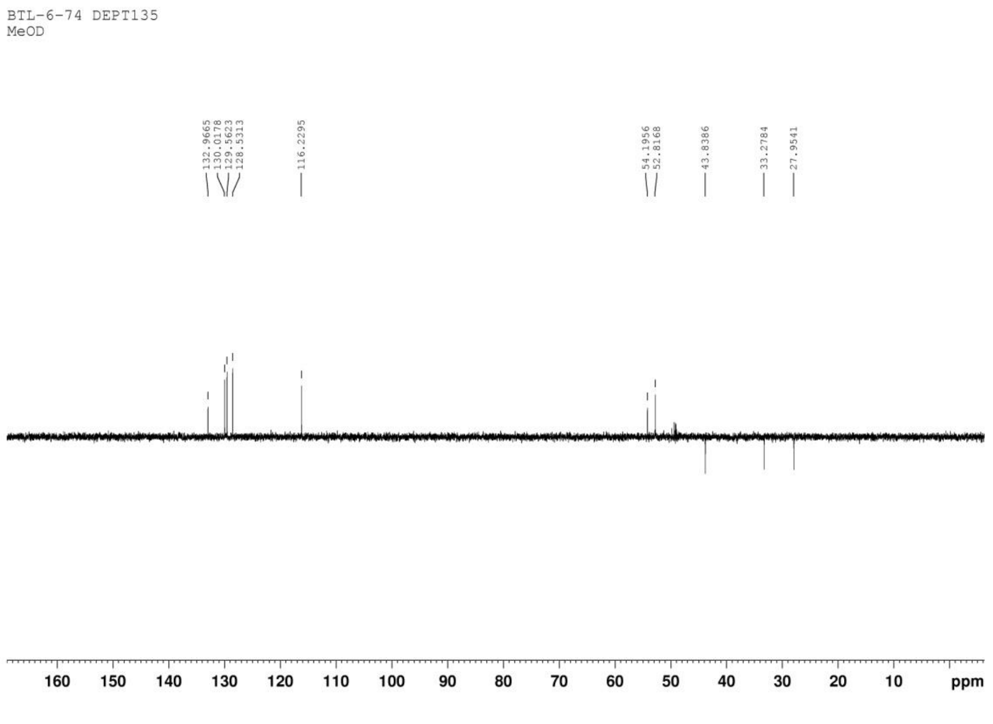

Figure S13 The DEPT 135 spectrum of compound **2**

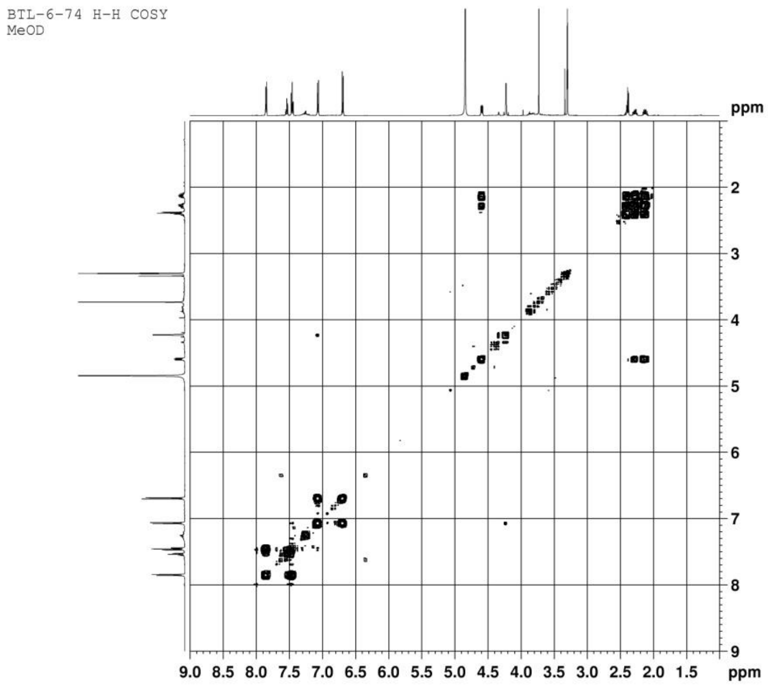

Figure S14 The ^{1}H-^{1}H COSY spectrum of compound **2**

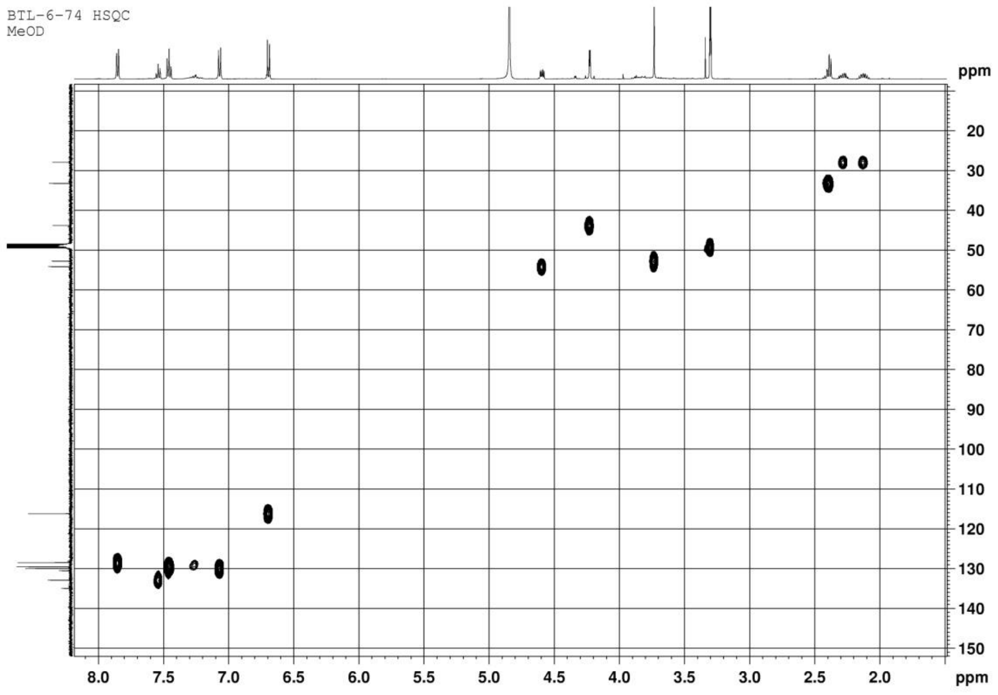

Figure S15 The HSQC spectrum of compound **2**

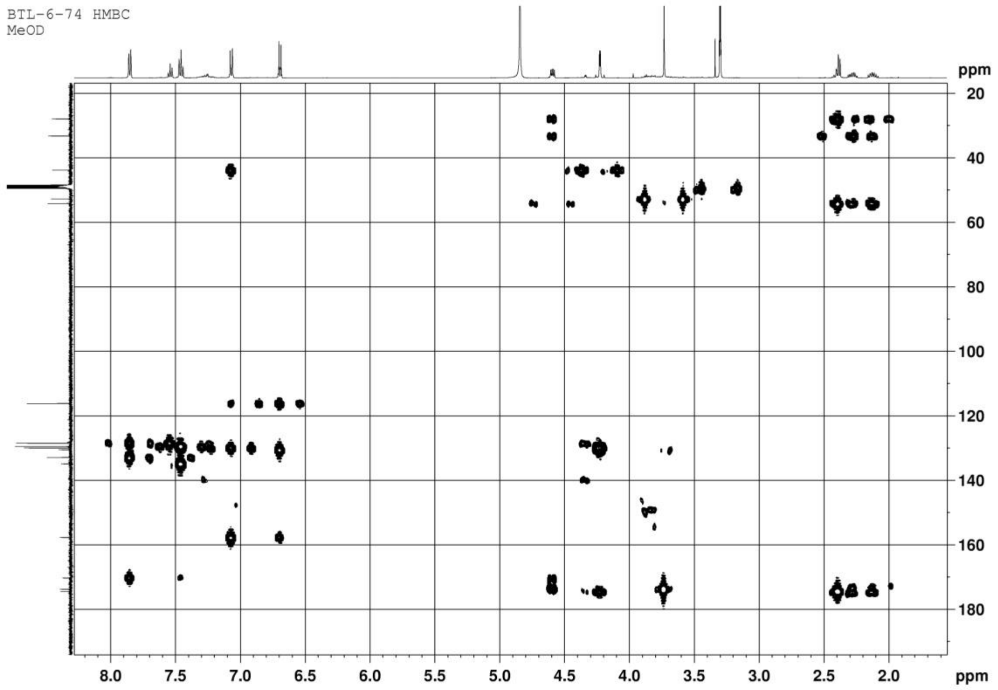

Figure S16 The HMBC spectrum of compound **2**

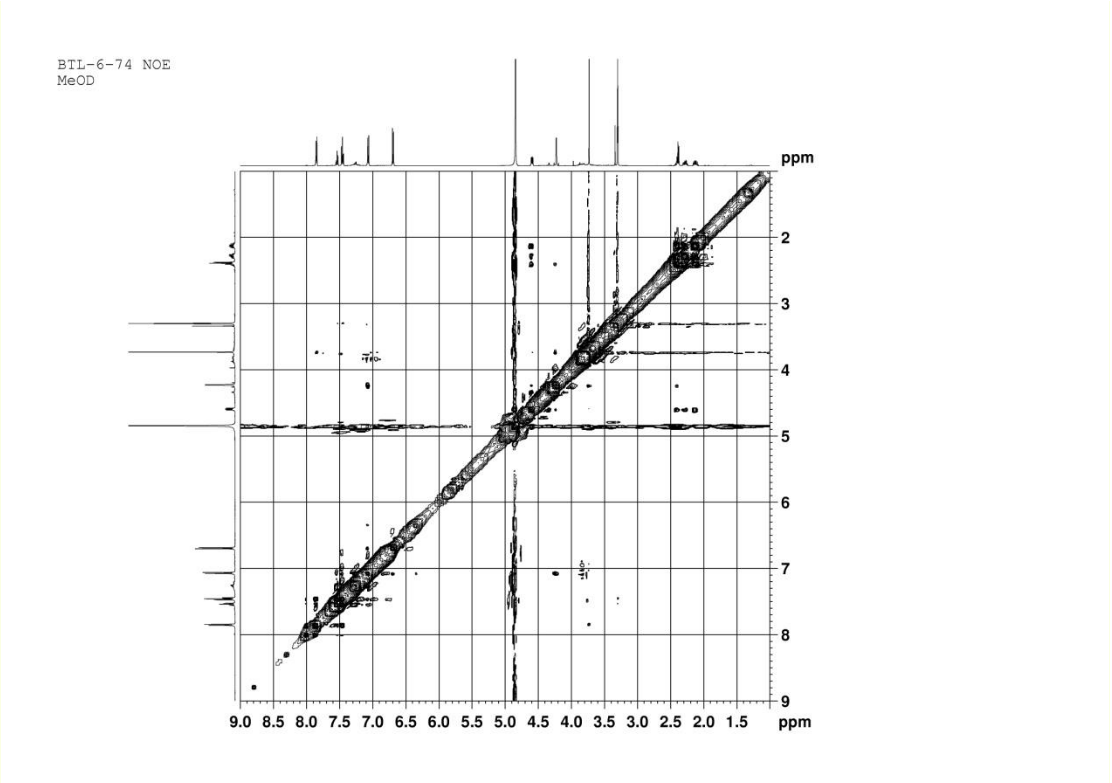

Figure S17 The NOESY spectrum of compound **2**

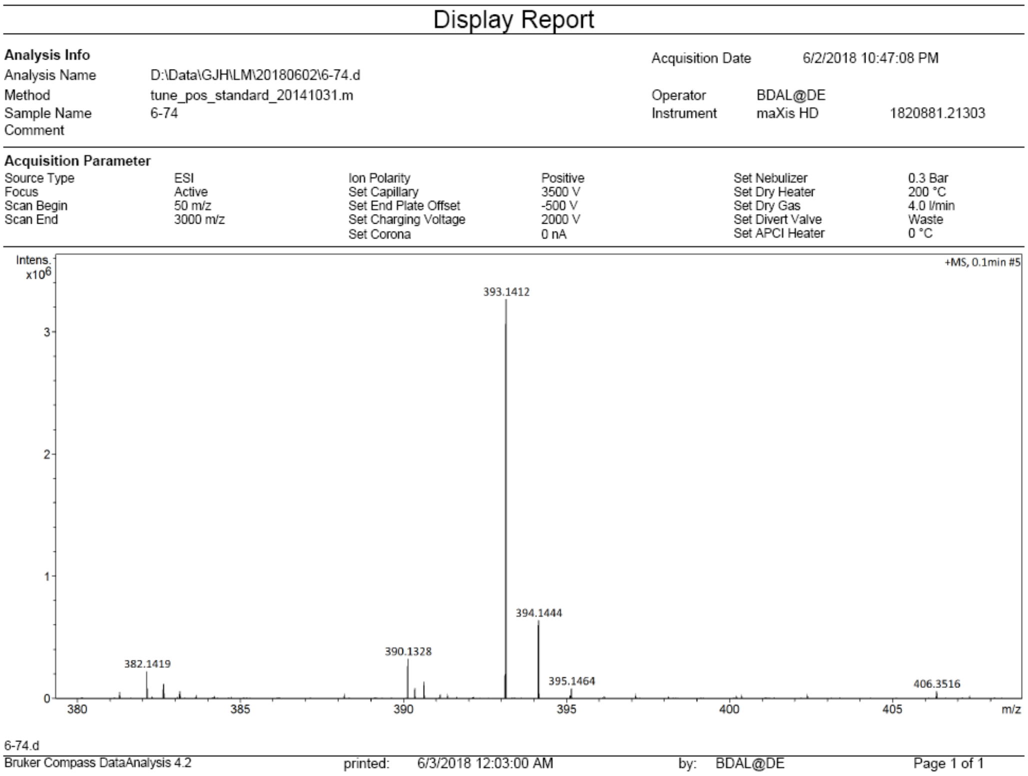

Figure S18 The HRESIMS spectrum of compound **2**

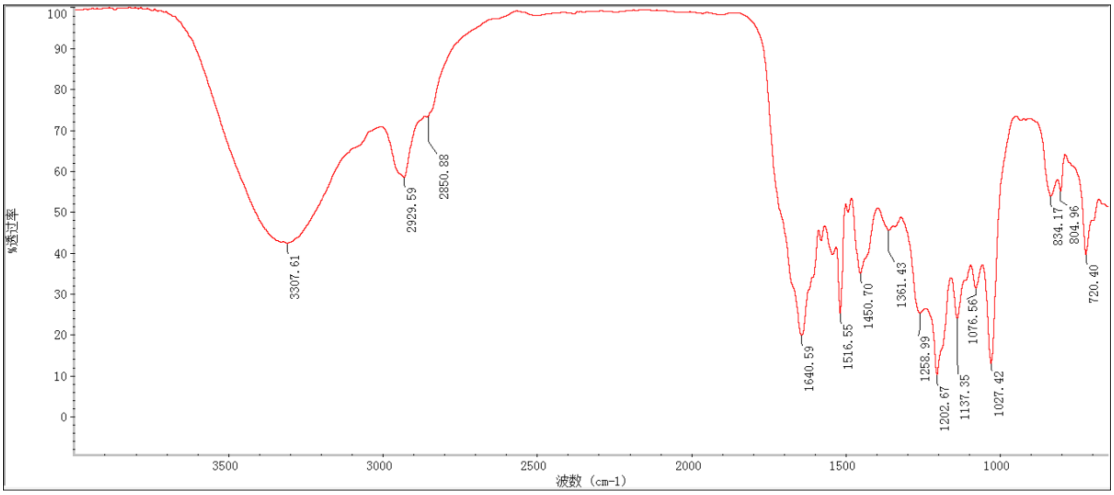

Figure S19 The IR spectrum of compound **2**

Scan Graph

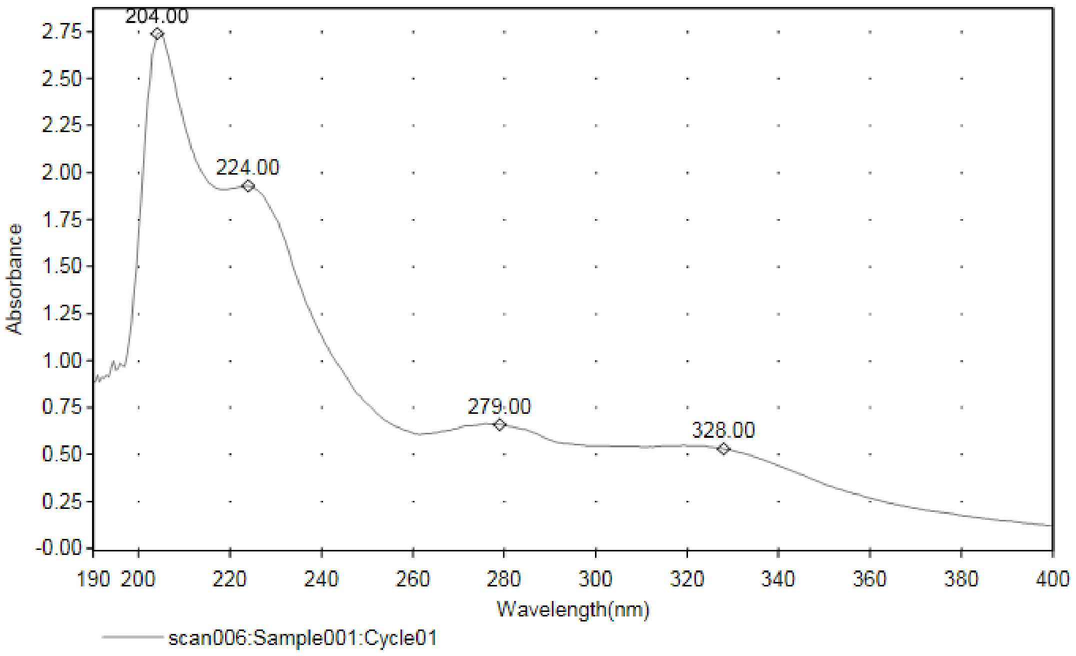

Results Table - scan006,Sample001,Cycle01

nm	A
204.00	2.739
224.00	1.929
279.00	.659
328.00	.530

Manual Method
Report Values at 4 Wavelengths
204.00 nm 224.00 nm 279.00 nm 328.00 nm
Sort By Wavelength

Figure S20 The UV spectrum of compound **2**

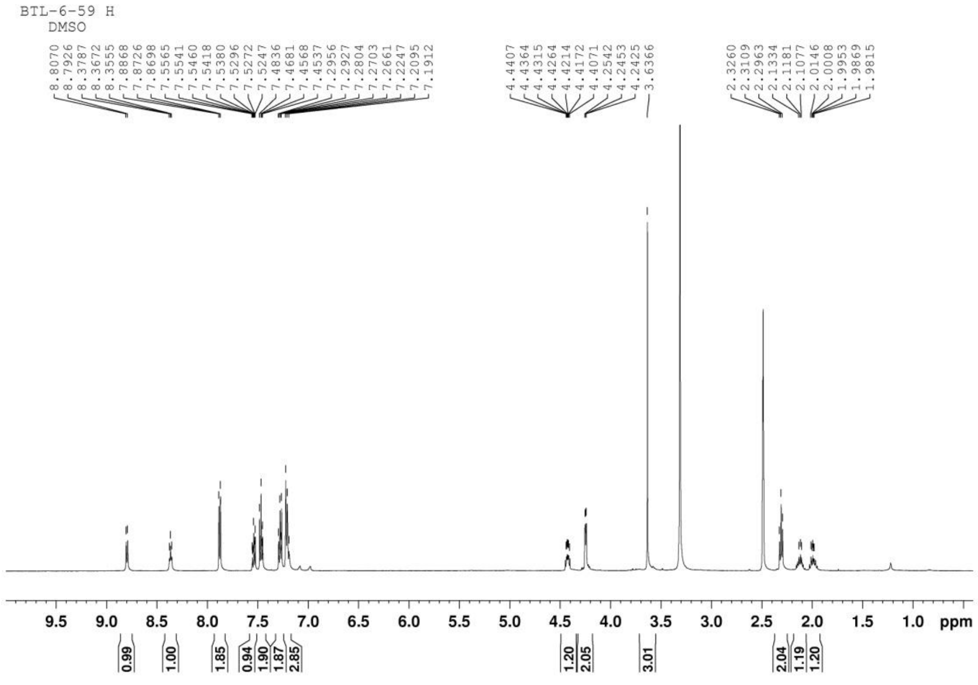

Figure S21 The ^{1}H-NMR spectrum of compound **3**（in DMSO-d_6）

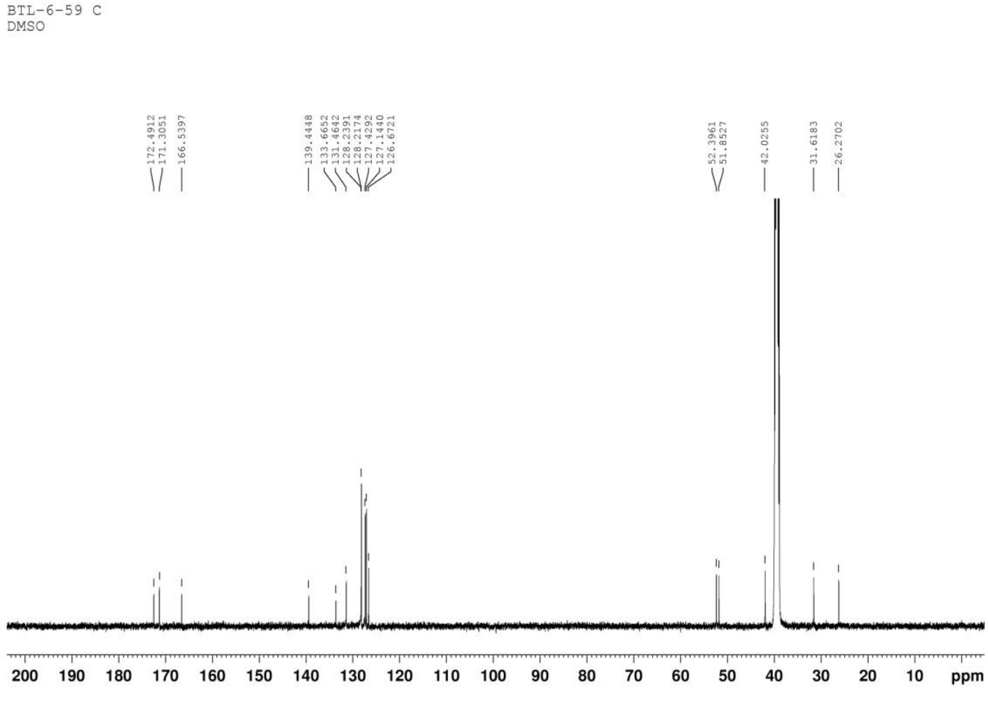

Figure S22 The ^{13}C-NMR spectrum of compound（in DMSO-d_6）

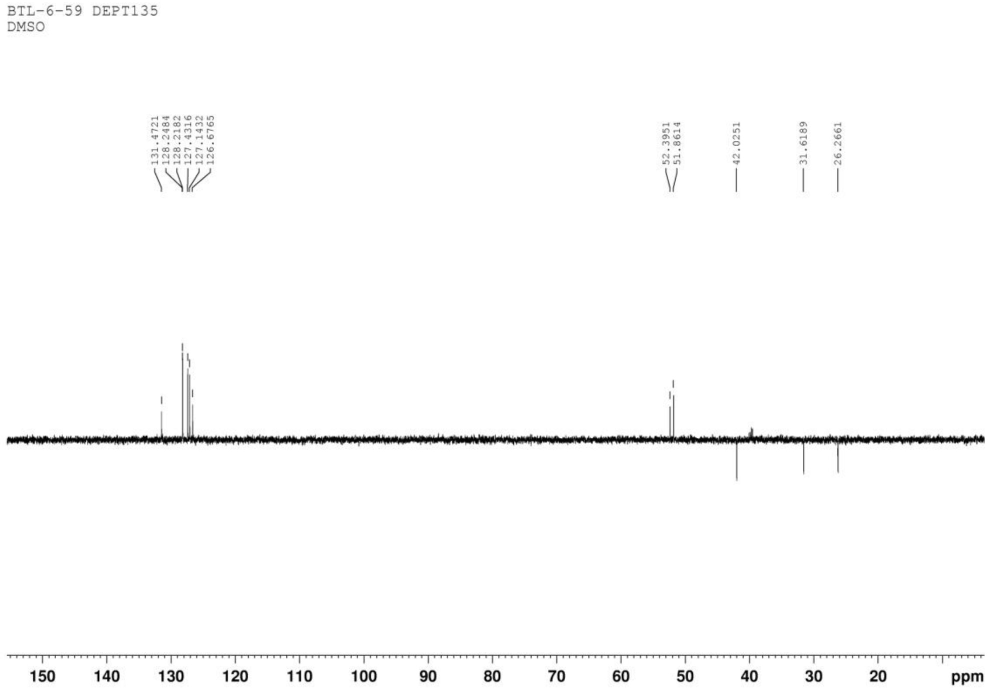

Figure S23 The DEPT 135 spectrum of compound **3**（in DMSO-d_6）

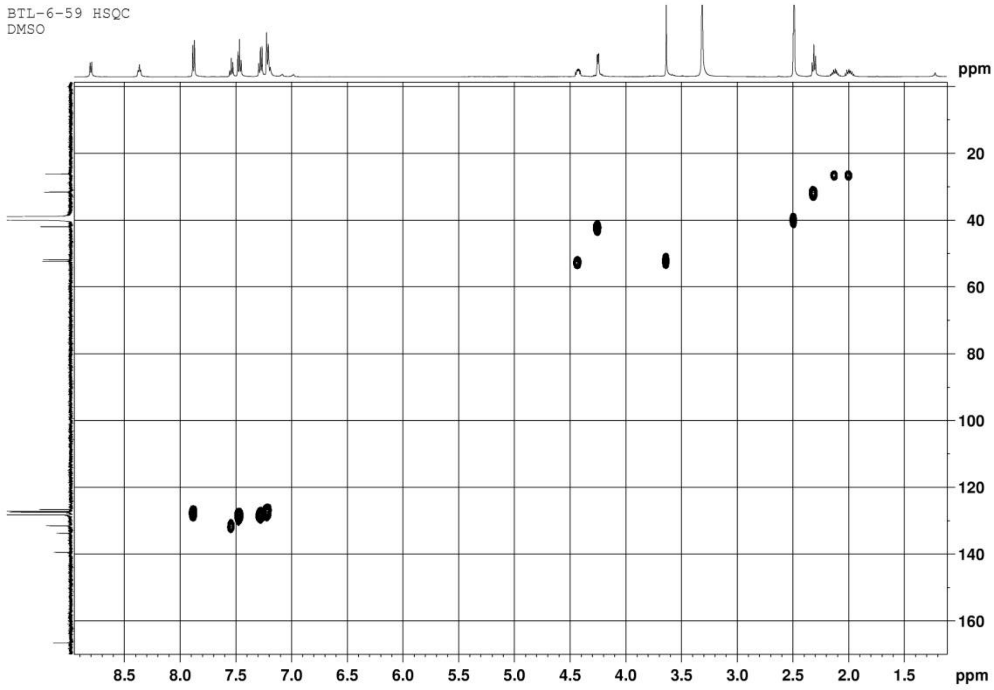

Figure S24 The HSQC spectrum of compound **3**

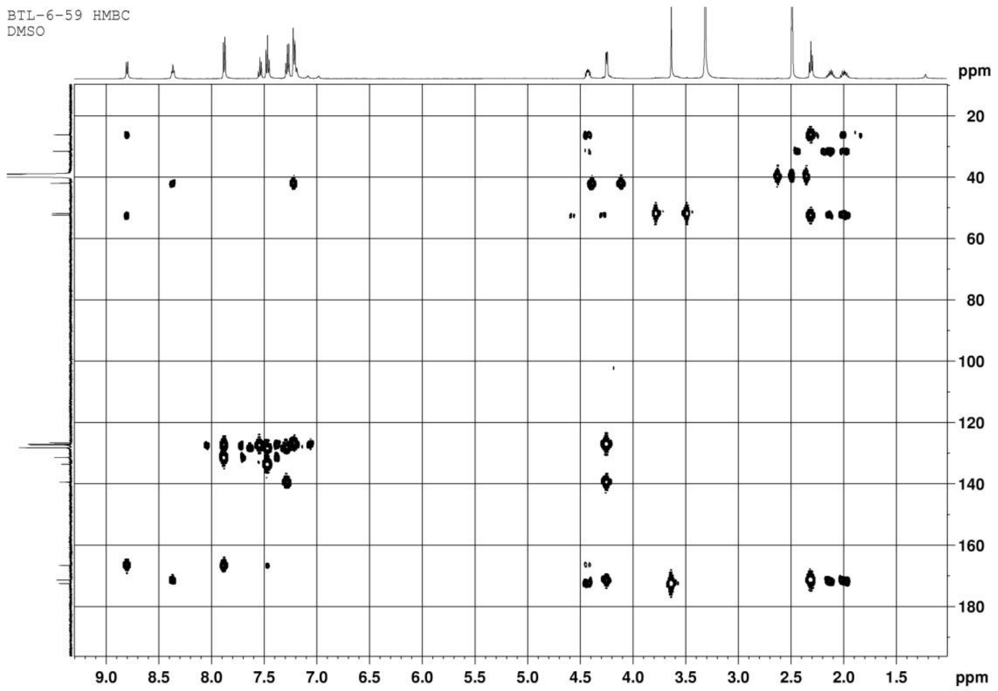

Figure S25 The HMBC spectrum of compound **3**

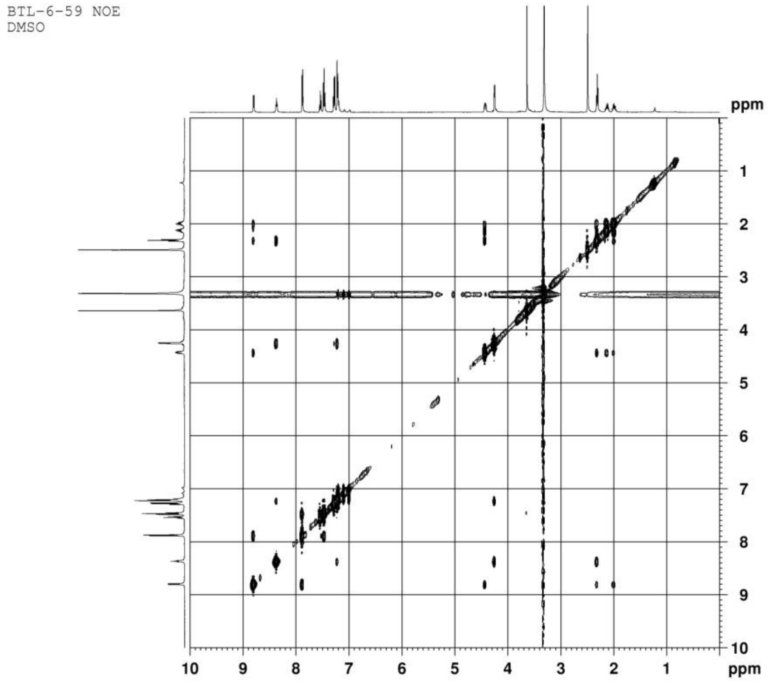

Figure S26 The NOESY spectrum of compound **3**

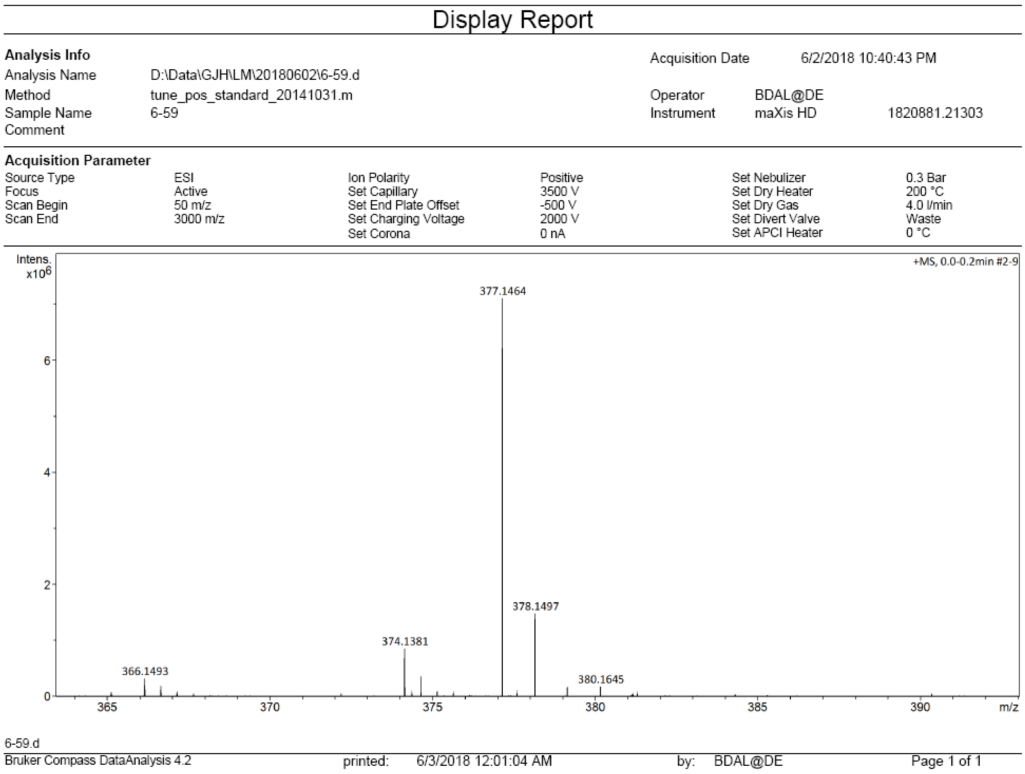

Figure S27 The HR-ESI-MS spectrum of compound **3**

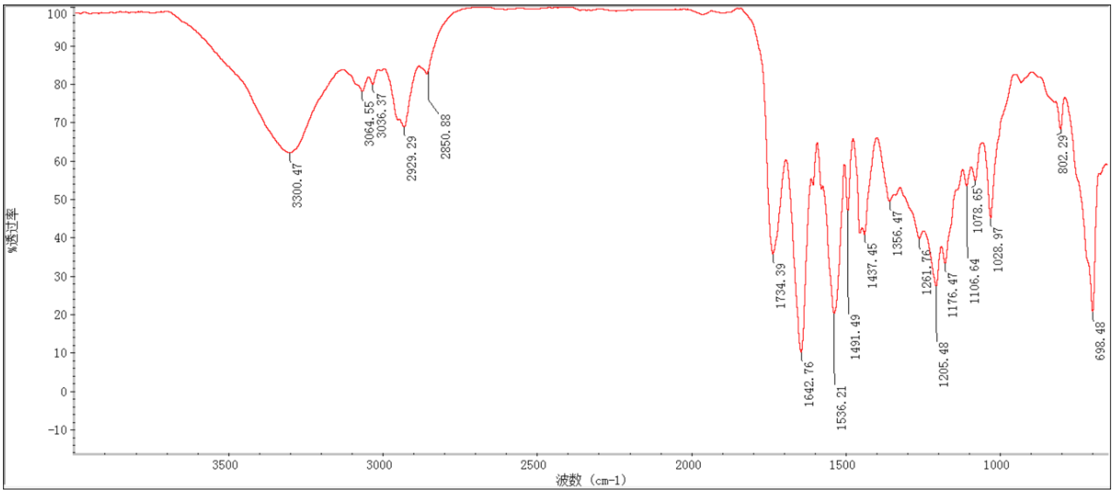

Figure S28 The IR spectrum of compound **3**

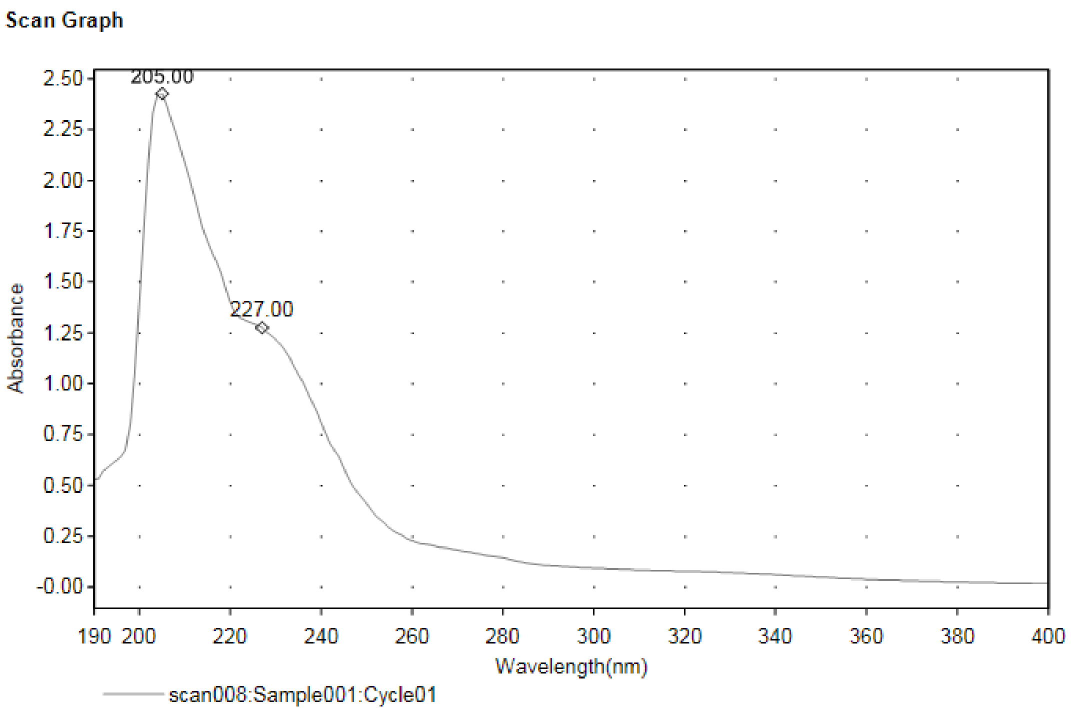

Results Table - scan008,Sample001,Cycle01

nm	A	**Manual Method**
205.00	2.425	Report Values at 2 Wavelengths
227.00	1.275	205.00 nm 227.00 nm
		Sort By Wavelength

Figure S29 The UV spectrum of compound **3**

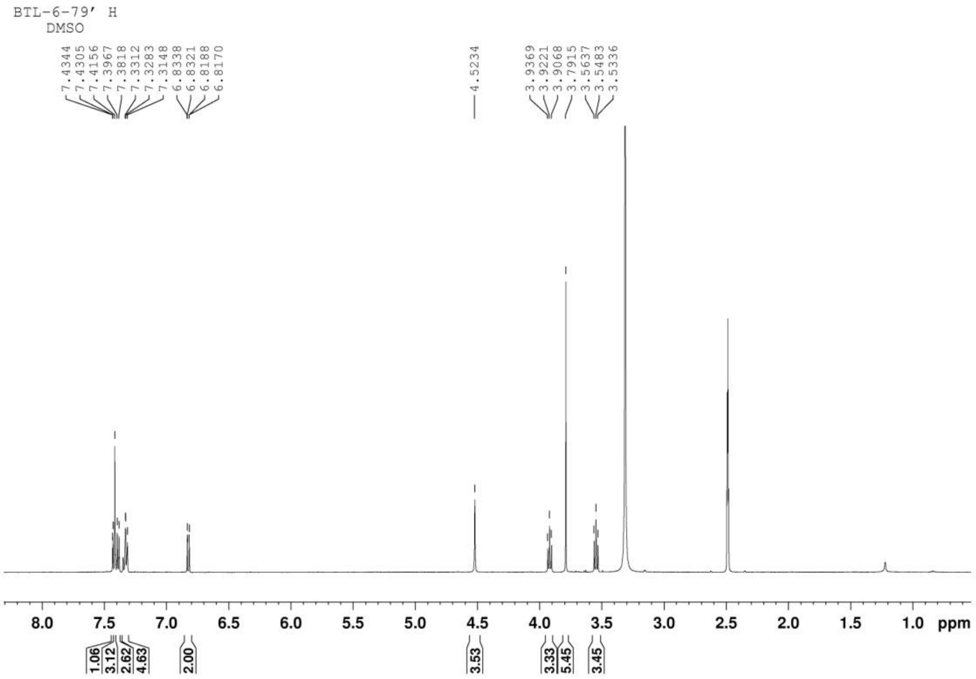

Figure S30 The ^{1}H-NMR spectrum of compound **4**（in DMSO-d_6）

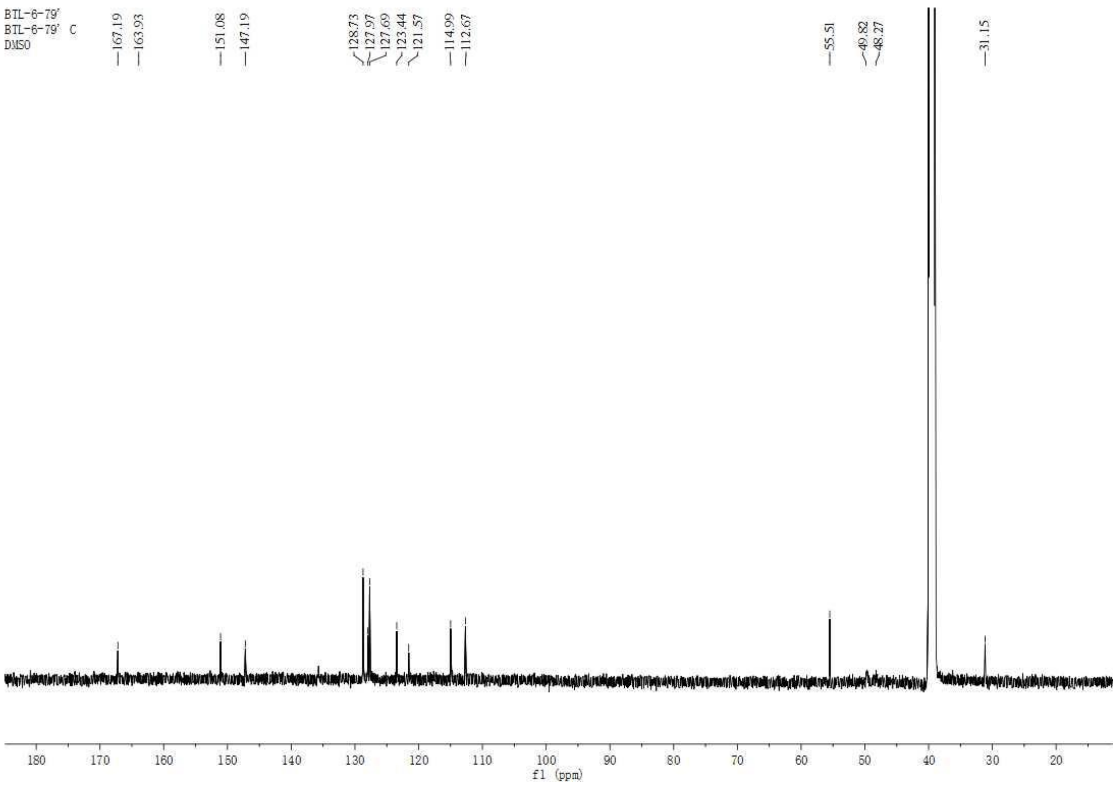

Figure S31 The ^{13}C-NMR spectrum of compound **4**（in DMSO-d_6）

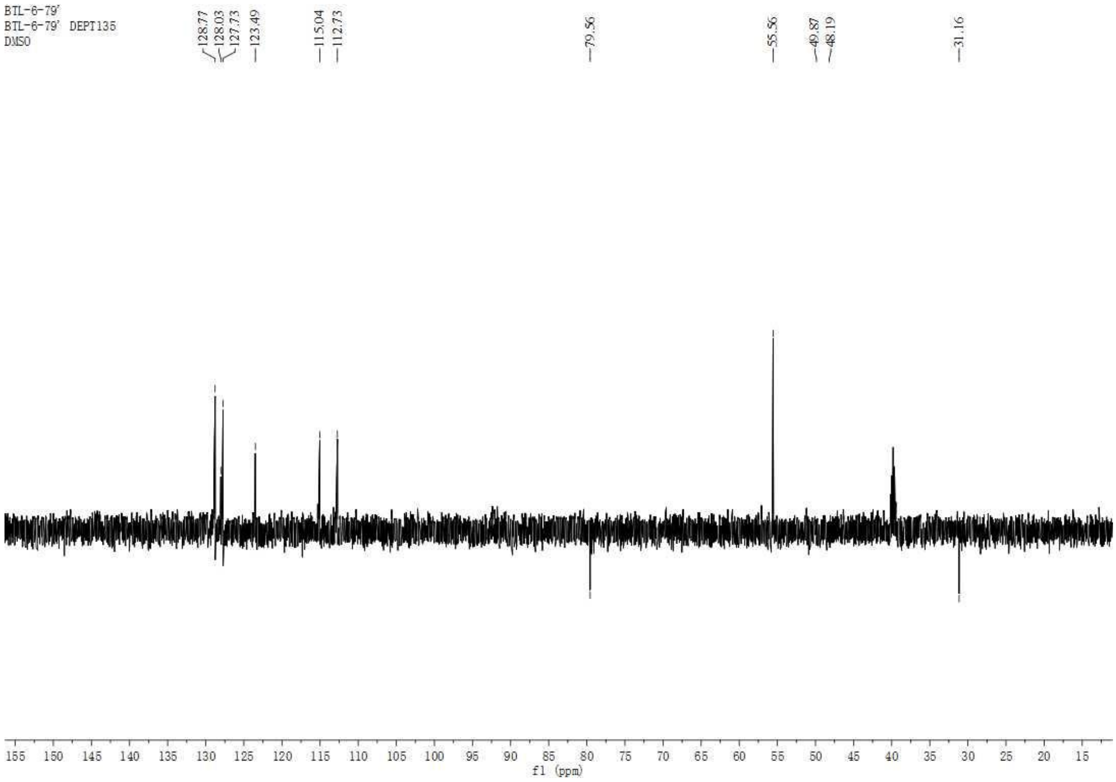

Figure S32 The DEPT 135 spectrum of compound **4**

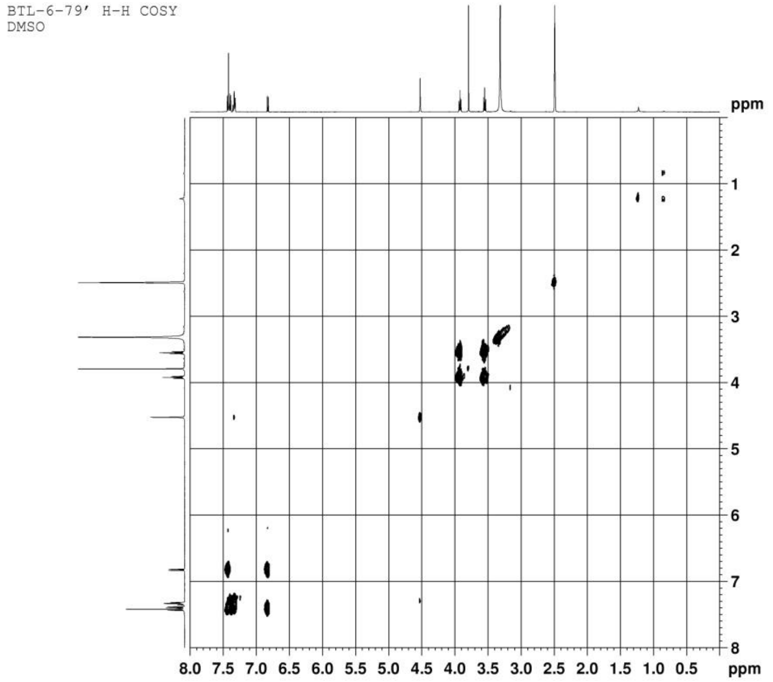

Figure S33 The ^{1}H-^{1}H COSY spectrum of compound **4**

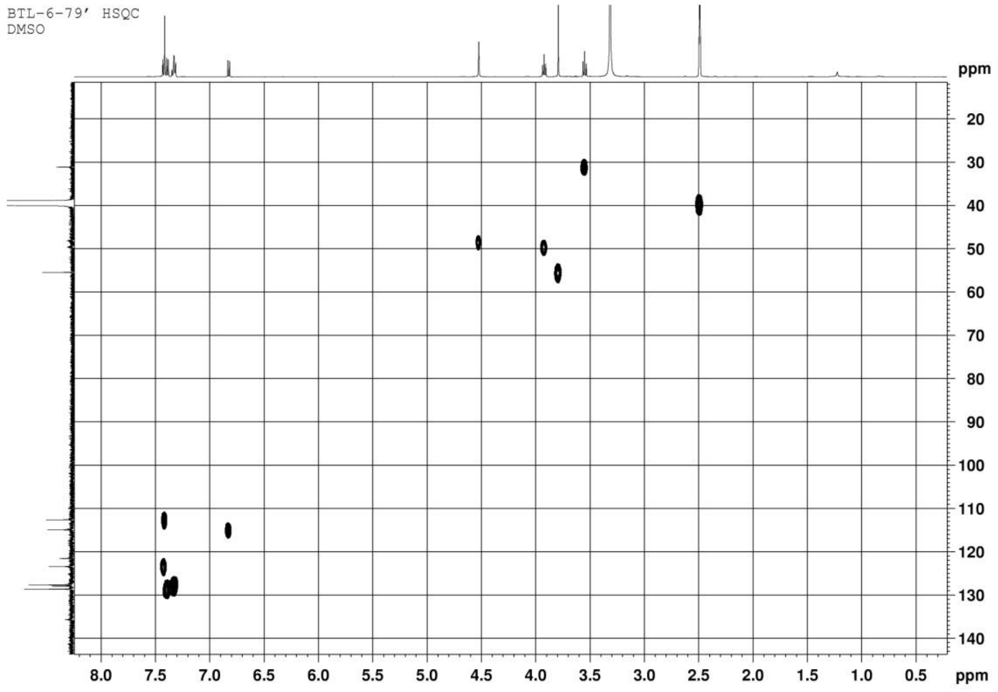

Figure S34 The HSQC spectrum of compound **4**

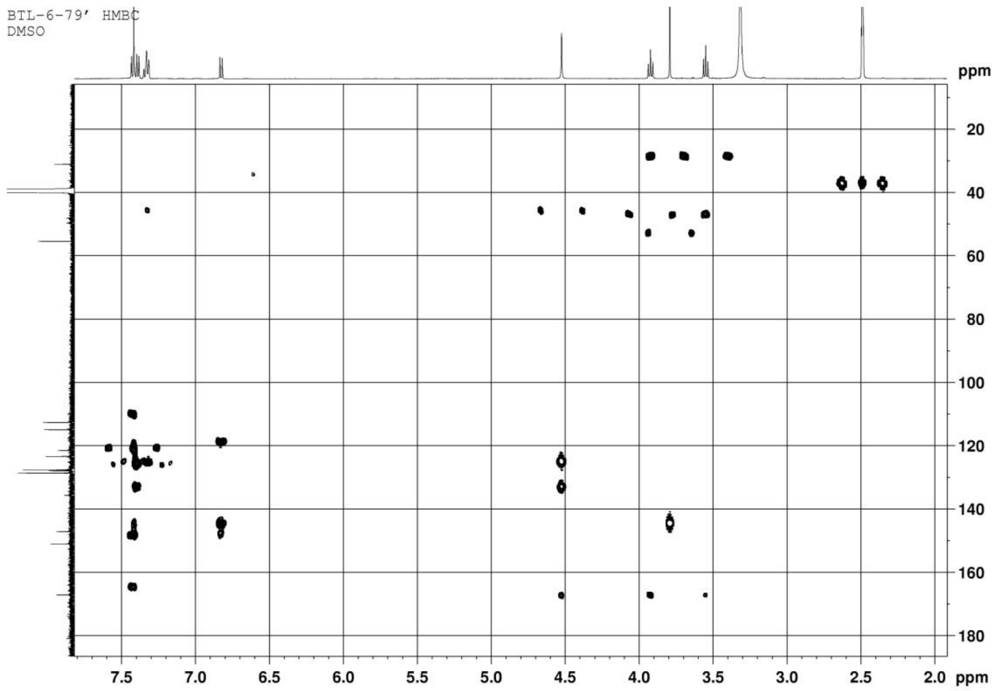

Figure S35 The HMBC spectrum of compound **4**

(Figure S31)

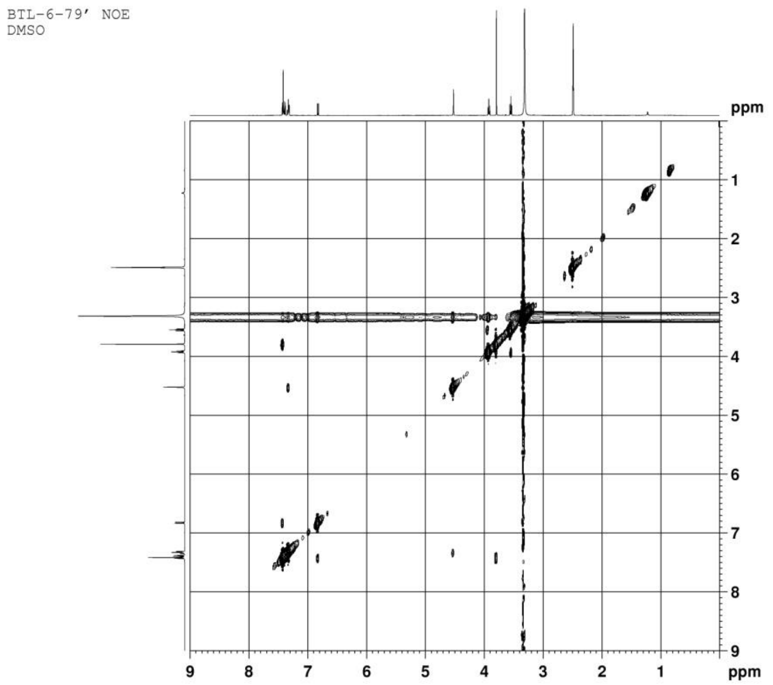

Figure S36 The NOESY spectrum of compound **4**

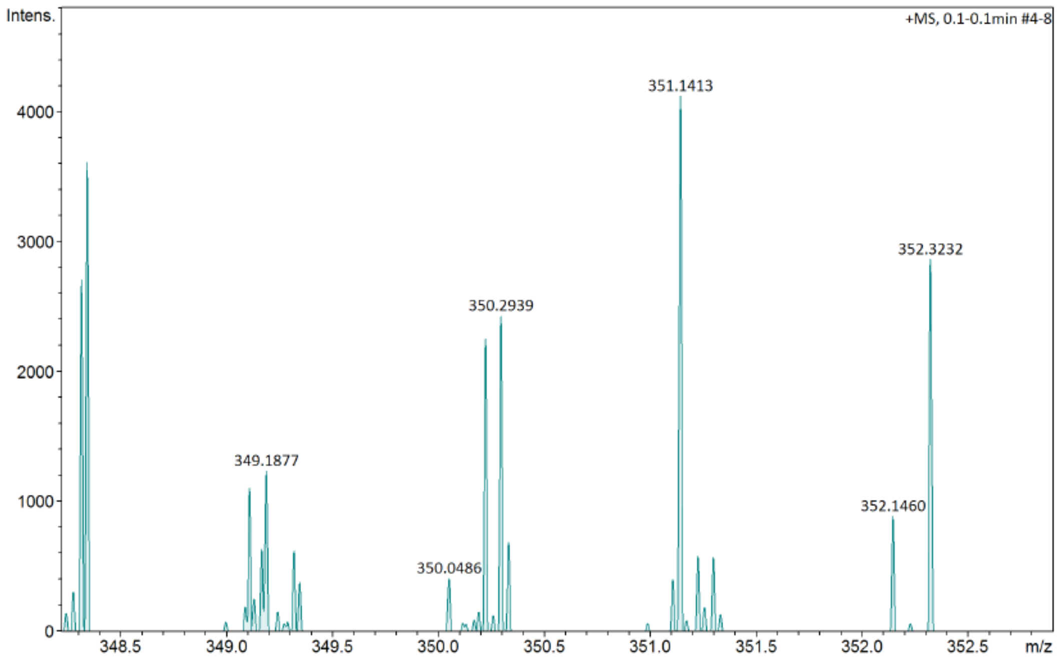

Figure S37 The HRESIMS spectrum of compound **4**

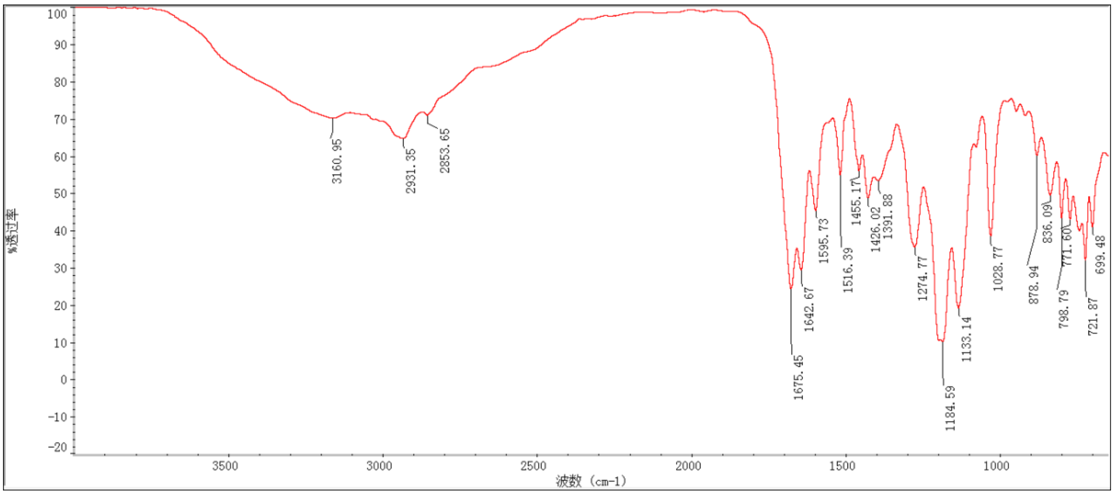

Figure S38 The IR spectrum of compound **4**

Scan Graph

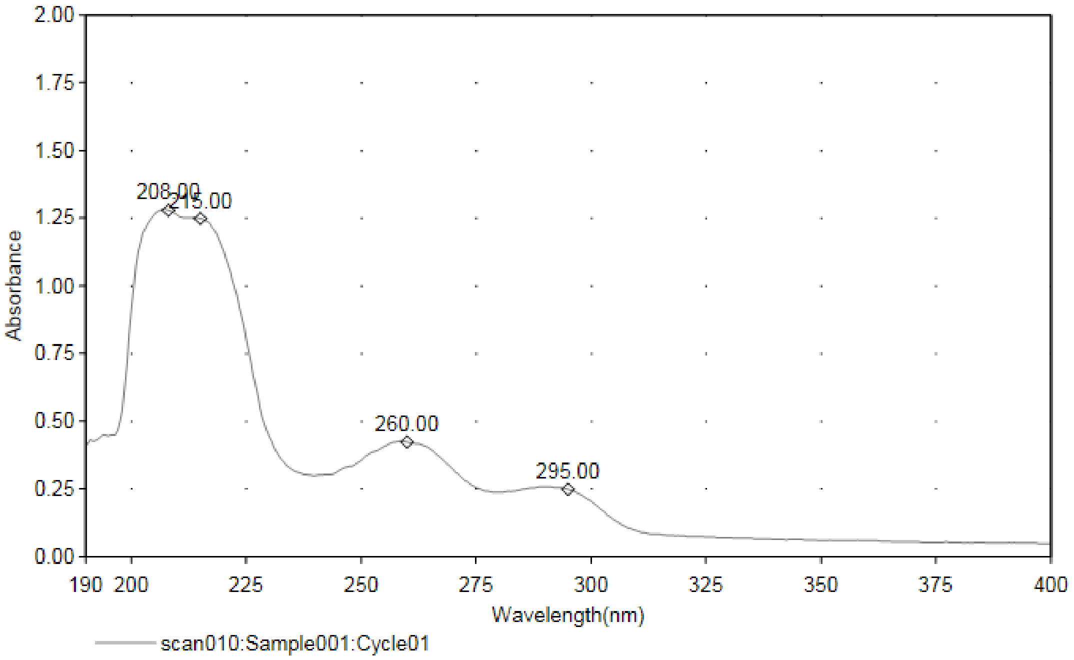

Results Table - scan010,Sample001,Cycle01

nm	A	**Manual Method**
208.00	1.279	Report Values at 4 Wavelengths
215.00	1.247	208.00 nm 215.00 nm 260.00 nm 295.00 nm
260.00	.423	Sort By Wavelength
295.00	.248	

Figure S39 The UV spectrum of compound **4**
